# Mixed neuroendocrine-non-neuroendocrine carcinoma of gallbladder: case report

**DOI:** 10.1186/s12957-019-1598-4

**Published:** 2019-03-22

**Authors:** Adam Skalický, Lucie Vištejnová, Magdaléna Dubová, Tomáš Malkus, Tomáš Skalický, Ondřej Troup

**Affiliations:** 1 Department of Surgery and Biomedical Center, Faculty of Medicine and University Hospital in Pilsen, Charles University in Prague, 304 60 Pilsen, Czech Republic; 2 Biomedical Center, Faculty of Medicine in Pilsen, Charles University, 304 60 Pilsen, Czech Republic; 3 Šikl’s Department of Pathology, Faculty of Medicine and University Hospital in Pilsen, Charles University, 305 99 Pilsen, Czech Republic; 4 Department of Imaging Methods, University Hospital in Pilsen, 304 60 Pilsen, Czech Republic; 5 Faculty of Medicine in Pilsen, Charles University, 301 00 Pilsen, Czech Republic

**Keywords:** Mixed neuroendocrine-non-neuroendocrine tumors, MINEN, Gallbladder cancer, Somatostatin analogues

## Abstract

**Background:**

Mixed neuroendocrine-non-neuroendocrine tumors (MINEN) of the gallbladder are extremely rare; indeed, the English expert literature reports a mere handful of case reports and case series on this topic. According to the WHO classification of 2010, MINEN are considered to be tumors consisting of two major components, neuroendocrine and non-neuroendocrine, each of which hosts at least 30% of the total cellular population. To date, the etiology and pathogenesis of MINEN have not been precisely determined and the non-specific symptoms generally result in late diagnosis (mainly in the terminal stages of the condition) and contribute to the generally poor prognosis. As far as the management of the disease is concerned, radical surgery plays a crucial role; however, the significance of surgical debulking and biological therapy applying somatostatin analogues has not yet been determined.

**Case presentation:**

A 56-year-old female was referred to our department for a rapidly progressing tumor in the subhepatic area along with the infiltration of S5 and S6 liver segments. With regard to preoperative findings, the tumor appeared as operable, although, during the surgery, an extensive involvement of the hepatoduodenal ligament by the tumor through the lymph nodes was revealed. Due to acute perioperative bleeding from the necrotic tumor, we decided to perform modified resection. Histologically, the tumor was confirmed as MINEN of gallbladder, where the neuroendocrine component was dominant over the non-neuroendocrine component. Six weeks after the discharge, the patient underwent a follow-up CT revealing large recurrence of the disease. Thereafter, the patient was started on systemic therapy with etoposide and carboplatin in combination with somatostatin analogues. Thirteen months after the surgery, the patient is in good clinical condition, and while a recently performed PET/MRI scan revealed a hepatic lesion and hilar lymphadenopathy in full regression, there was a spread of small peritoneal and pleural metastases. The patient remains in the follow-up care.

**Conclusions:**

The occurrence of mixed neuroendocrine-non-neuroendocrine neoplasms is extremely rare. Radical surgery remains the only potentially effective approach to the cure of this disease. The role of biological therapy and debulking in the management of the disease has not yet been precisely defined. In our experience, both of these methods have the potential to positively influence overall survival rates and the postoperational quality of life of patients.

## Background

Neuroendocrine tumors (NET) of the gastrointestinal tract constitute a heterogeneous group of disorders that differ from each other depending on the site of origin and the degree of tumor differentiation. The current terminology used in the 2010 *WHO Classification of Tumors of the Digestive System* is based on NET assessment using morphological criteria and proliferative activity of tumor cells [1, 2] The biological properties of the tumor are determined by the degree of differentiation (grading) and the extent of progression (staging) related to the localization of the tumor, as determined according to the 2009 TNM Classification of Malignant Neoplasms [3].

Mixed neuroendocrine-non-neuroendocrine tumors (MINEN) is a rare type of tumor formed by two components, a non-neuroendocrine component (most often adenoma/adenocarcinoma) and a neuroendocrine tumor (NET G1, NET G2, NEC), where it was arbitrarily determined that each of these components must represent at least 30% of the tumor. The individual components of the tumor can be separated from each other, or in other cases they are merged into each other diffusely. Grading in the case of such lesions is described separately for the two components, and the total grade of the tumor is higher of two values; the prognosis is also determined by the higher value, i.e., by the biologically dominant component of the tumor. Usually, the lesions are highly aggressive and quickly metastasizing [2, 4, 5].

MINEN of the gallbladder and the extrahepatic biliary tract are extremely rare lesions; English expert literature reports 22 case reports or case series (Table 1) [6–22]. These are malignant tumors, more aggressive than NET, and their biological behavior can be predicted to some extent based on size (tumors > 2 cm often spreading by infiltration to the liver tissue and metastasizing into regional lymph nodes), localization (tumors of the extrahepatic biliary tract are more aggressive), depth of invasion, and presence of perineural propagation, which are associated with a worse prognosis. No precise rules have been determined for staging NETs of the gallbladder and biliary tract due to a very low prevalence of these lesions, and it is recommended that adenocarcinoma staging system be used for this region [6, 23, 24].

**Table 1:** The 22 case reports or case series

Number	Author [ref.]	Age/sex	Neuroendocrine component	Epithelial component
1	Acosta et al. [6]	55/F	NEC, LCNEC	Intestinal-type adenocarcinoma
2	Al-Brahim and Albannai [7]	45/M	NEC, LCNEC	Adenocarcinoma
3	Harada et al. [8]	70/F	NEC, small cell carcinoma	Well-differentiated adenocarcinoma
4	Harada et al. [8]	70/F	NEC, LCNEC	Well-differentiated papillary adenocarcinoma
5	Harada et al. [8]	70/F	NET G2, atypical carcinoid	Well-differentiated adenocarcinoma
6	Harada et al. [8]	60/F	NEC, small cell carcinoma	Well-differentiated papillary adenocarcinoma
7	Harada et al. [8]	50/F	NEC, LCNEC	Well-differentiated adenocarcinoma
8	Iype et al. [9]	85/M	NEC, LCNEC	Adenocarcinoma
9	Jung et al. [10]	54/F	NEC, LCNEC	Adenosquamous carcinoma
10	Kamboj et al. [11]	65/F	Unknown	Unknown
11	Kim et al. [12]	Unknown/M	Unknown	Unknown
12	Kim et al. [12]	Unknown/M	Unknown	Unknown
13	Lin et al. [13]	43/F	NEC, small cell carcinoma	Adenocarcinoma
14	Liu et al. [14]	63/F	NEC, LCNEC	Moderately differentiated adenocarcinoma
15	Meguro et al. [15]	54/F	NEC, LCNEC	Poorly differentiated biliary type adenocarcinoma
16	Mondolfi et al. [16]	48/F	NEC, LCNEC	Papillary adenocarcinoma
17	Noske and Pahl [17]	81/F	Poorly differentiated LCNEC	Moderately differentiated adenocarcinoma
18	Oshiro et al. [18]	55/F	NEC, small cell carcinoma, LCNEC	Papillary adenocarcinoma, tubular adenocarcinoma
19	Sato et al. [19]	68/F	NEC, LCNEC	Well-differentiated tubular adenocarcinoma
20	Shimiziu et al. [20]	58/M	NEC, small cell carcinoma	Well-differentiated tubular adenocarcinoma
21	Shintaku et al. [21]	80/M	NET G2	Tubular adenocarcinoma, squamous cell carcinoma, osteosarcoma
22	Song et al. [22]	55/F	NEC, small cell carcinoma	Adenocarcinoma

*NEC* neuroendocrine carcinoma; *LCNEC* large cell neuroendocrine carcinoma; *NET G2* neuroendocrine tumor, grade 2; *F* female; *M* male

## Case presentation

**Fig. 1:**
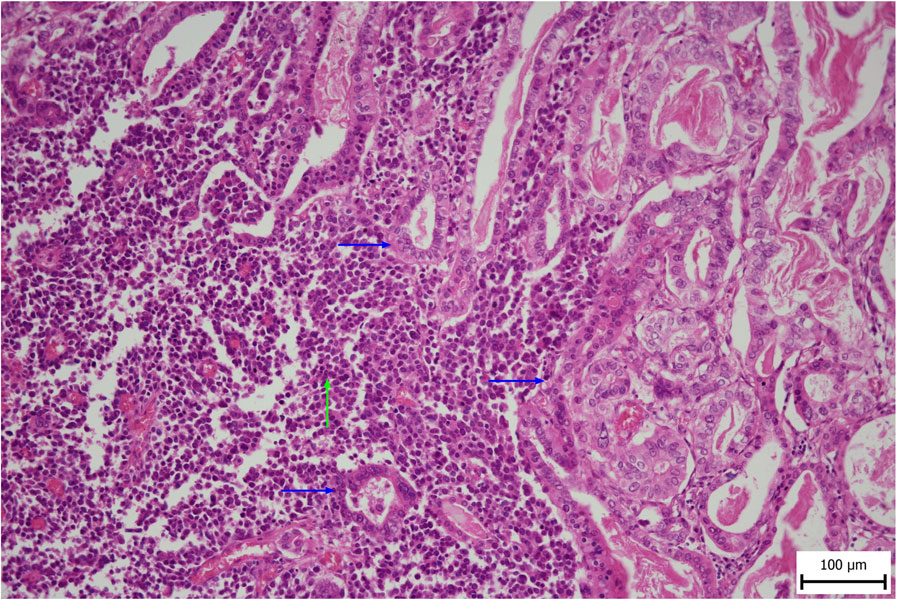
Intimate relation of the adenocarcinomatous component (blue arrows) and the neuroendocrine small cell component (green arrows) in the mixed neuroendocrine carcinoma (hematoxylin eosin, × 200)

**Fig. 2:**
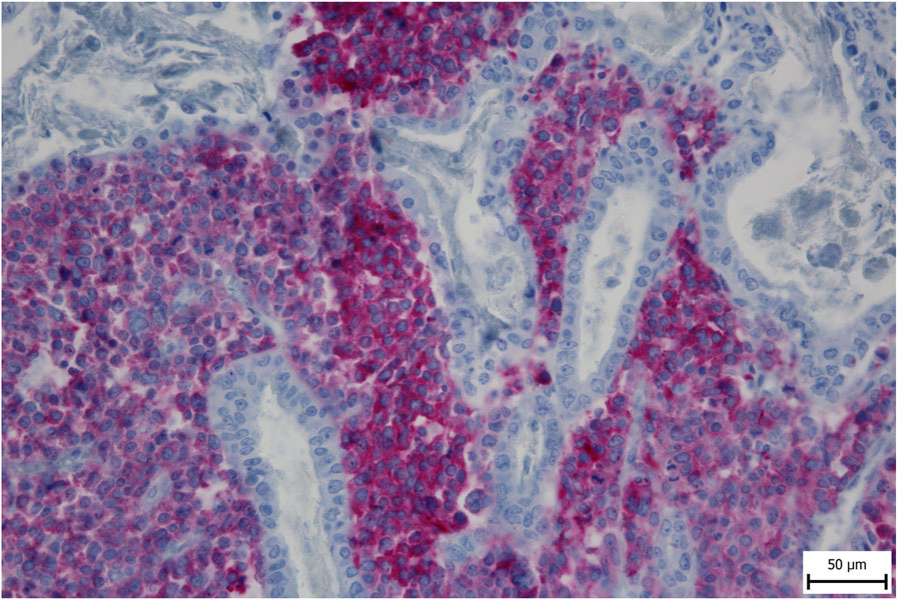
The neuroendocrine component is positively dyed by an anti-synaptophysin antibody whereas the adenocarcinoma’s glands are negative (synaptophysin, × 400)

**Fig. 3:**
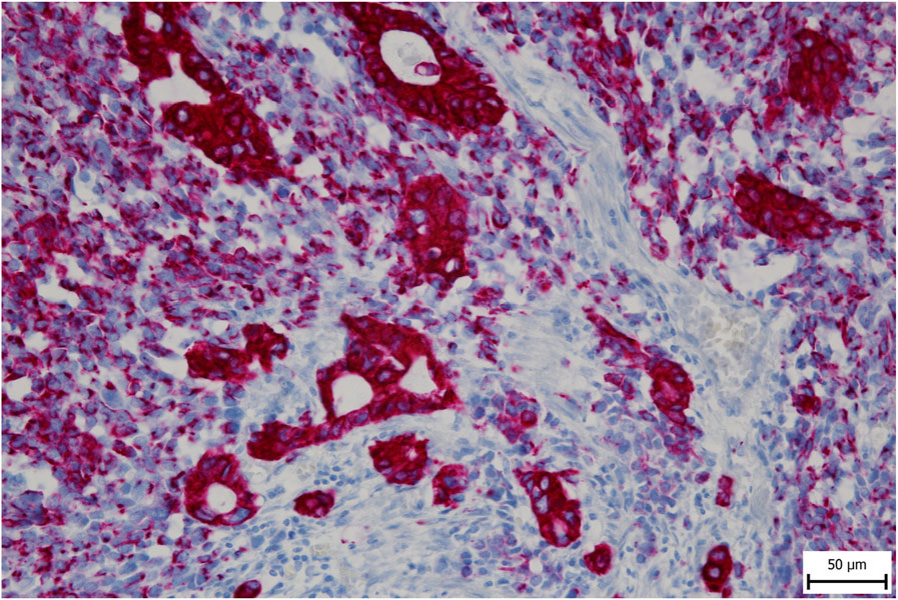
More pronounced positivity of cytokeratins in tumor glands in comparison with the expression in neuroendocrine cells (CAM5.2, × 400)

**Fig. 4:**
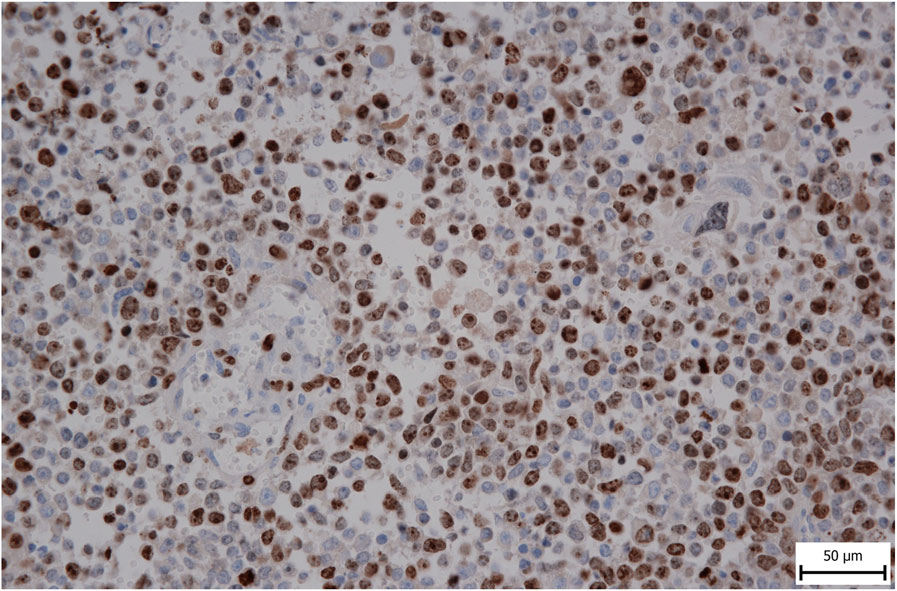
High proliferation index of the small cell neuroendocrine component (MIB-1, × 400)

**Fig. 5:**
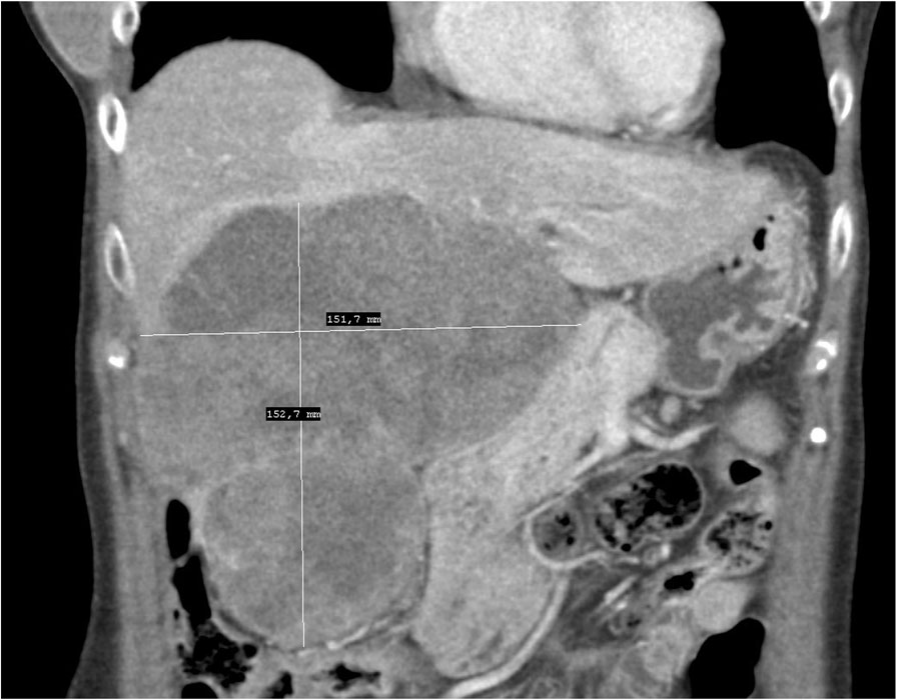
Preoperative CT scan

**Fig. 6:**
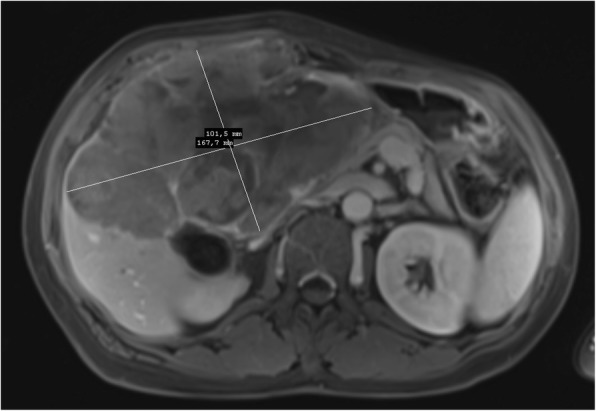
Preoperative MRI scan

**Fig. 7:**
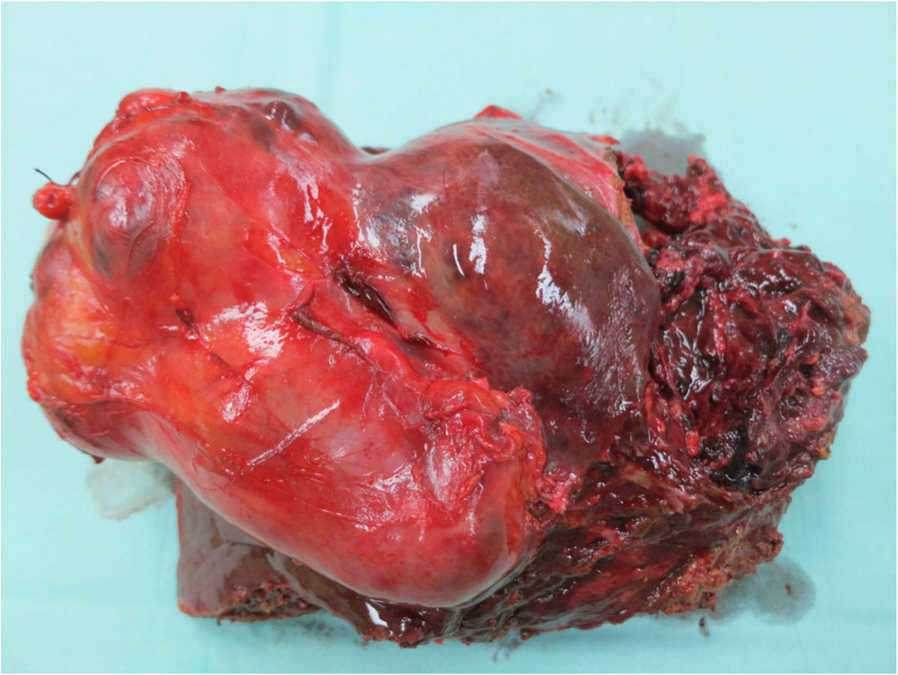
Resected specimen of the liver with evident dual tumorous component

A 56-year-old female was referred to our department from another facility in the patient’s area for a rapidly progressing tumor in the gallbladder and liver area. The patient reported several-month right upper quadrant pain and 4-kg weight loss over the past year. There was no laboratory sign of obstructive jaundice at the day of admission. Preoperative CT and MR scan (Figs. 5 and 6) of the liver was performed, and the patient was diagnosed with a tumor in the gallbladder area with a relatively massive infiltration of the S5 and S6 liver segments and extensive regions of necrosis. Given the potentially resectable lesion according to preoperative imaging, exploratory laparotomy was indicated to attempt radical resection. During the exploration, a voluminous tumor was found attached to the peritoneum. Intraoperative ultrasound was performed and revealed a tumor originating from the gallbladder bed area and reaching up to the area of the hepatic hilum and extensive involvement of the hepatoduodenal ligament by the tumor through the lymph nodes. The tumor was classified as inoperable due to this finding. But during the exploration, however, a rupture of the fragile tumor occurred with massive eruption of the necrotic mass and the gallbladder content into the abdominal cavity, accompanied by bleeding of the liver parenchyma. We decided that the condition could only be managed by attempting modified resection. We performed cholecystectomy and non-anatomical resection of hepatic segments S5 and S6 and partial resection of S4 without lymphadenectomy as a debulking operation (Fig. 7). The course of hospitalization was uncomplicated, and the patient was discharged to home care on postoperative day 9. Histologically, the tumor was confirmed as MINEN of gallbladder (Figs. 1, 2, and 3), and its non-neuroendocrine component had the character of moderately differentiated tubular gall bladder adenocarcinoma, while the neuroendocrine component had the appearance of small cell carcinoma and was dominant, accounting for more than 65% of the viable tumor. The neuroendocrine component contained extensive necrosis, with mitotic index 64/10 HPF and a proliferation index of 70% (Fig. 4). It was therefore obvious that the prognosis and the subsequent biological behavior would be influenced in particular by the neuroendocrine carcinoma component. Six weeks after the discharge, the patient underwent a follow-up CT scan prior to the initiation of systemic therapy, which revealed a large recurrence of the disease at the resection surface of the liver accompanied by hilar lymphadenopathy. The patient was started on systemic therapy with etoposide and carboplatin in combination with somatostatin analogues with very good radiological effect. We use this regimen as a standard in patients with MINEN of gastrointestinal tract with dominant neuroendocrine component, even with no somatostatin receptors staining available. Now the patient is almost a year after being diagnosed with a tumor, after completion of 6 cycles of adjuvant chemotherapy (carboplatin + etoposide) in combination with biological therapy, the long-acting somatostatin analogues. The patient is in good clinical condition, and while a recently performed PET/MRI scan revealed a hepatic lesion and hilar lymphadenopathy in full regression, there was a spread of small peritoneal and pleural metastases, with a solitary metastasis in Th9. The condition was evaluated as disease progression stage according to RECIST criteria, the patient remains in the follow-up care, and it is now 13 months after surgery (Figs. 5, 6, and 7).

## Discussion

Malignant tumors of the gallbladder are uncommon findings, accounting for about 0.5% of all malignancies [25]. Despite a relatively simple histology, a wide spectrum of malignant tumors may originate in the gallbladder (of epithelial, mesenchymal, or neuroendocrine origin). The most frequently occurring tumor is papillary adenocarcinoma found in 90% of cases [26]. In contrast, tumors with neuroendocrine differentiation account for less than 0.5% of gallbladder malignancies [27]. The origin of these malignancies is not yet completely understood, as a healthy gallbladder contains no neuroendocrine cells. The theory with the greatest support in the available literature assumes the formation of dysplasia and subsequent cancer growth in regions with metaplastic changes. It is well documented that particular regions of intestinal metaplasia are found in the chronic cholecystitis (most frequently in cholecystolithiasis), which already contain neuroendocrine cells [28]. It is also proved that radiotherapy and chemotherapy cause genomic instability and the number of neuroendocrine marker-positive cells increased following systematic treatment [29]. This suggests that MINEN might also develop during classic adenoma-carcinoma sequence [30, 31].

Recently, increasing importance is assigned to multipotent, progenitor cells persisting in the biliary tree, where they fulfill their role in regenerative processes [32]. These cells also have oncogenic potential and may serve as the basis for neuroendocrine tumors as well as for malignancies with multiple morphological phenotypes [16, 33].

MINEN, as well as other gallbladder tumors, do not have specific symptoms. The most common symptoms of the disease include non-specific abdominal pain, weight loss, anorexia, and obstructive jaundice (which is a sign of advanced disease). The basic imaging technique is computed tomography with intravenous administration of contrast agent. This examination is sometimes preceded by ultrasound of the abdominal compartment because of its affordability and non-invasive nature. Generally, gallbladder tumors initially have an appearance of polyps or local wall thickening and are often an incidental finding. Depending on the extent, advanced carcinoma may take the form of an intraluminal expansion of the gallbladder, diffuse thickening of the wall or a mass essentially replacing the gallbladder and further infiltrating the adjacent liver parenchyma. Findings from imaging techniques are dependent on many factors (tumor size, vascularization, degree of differentiation, histological structure, and the presence of secondary regressive changes). Smaller tumors are more homogenous, and the homogeneity decreases with increasing size, predominantly due to regressive changes. A separate chapter is the use of methods of nuclear medicine, such as SPECT, PET/CT, or PET/MRI. The most frequently used radiopharmaceutical is ^18^FDG, an indicator of increased glucose metabolism. A correctly indicated examination provides the specificity and sensitivity of almost 90–100% [34–36]. The rate of accumulation is directly proportional to the degree of dedifferentiation of cells and is associated with a poor prognosis [35]. FDG is the radiopharmaceutical of choice in MINEN type tumors which are highly dedifferentiated. This examination, however, may provide false-negative results in well-differentiated neuroendocrine tumors, for which glucose is not the only source of energy. In these cases, an examination can be selected that uses labeled somatostatin analogues [37].

The only potentially curative method is radical surgery achieving R0 resection. To date, there has been no consensus about the extent of the resection procedure. The selected type of surgery varies from simple cholecystectomy up to major hepatic resection. The type and extent of the resection procedure should reflect the stage of the disease. The standard procedure in early-stage disease is cholecystectomy, with lymphadenectomy of the lymph nodes of the hepatoduodenal ligament and en bloc resection of the hepatic parenchyma surrounding the gallbladder bed. In a locally advanced disease, hepatectomy is recommended. Systemic therapy can be used in the neoadjuvant and adjuvant setting [22]. Biological treatment with somatostatin analogues is a new modality for modifying the disease, in particular in cases of confirmed somatostatin receptor expression on the surface of tumor cells. The mechanism of action is multimodal. Somatostatin analogues have antiproliferative effects, inhibit angiogenesis, and have a pro-apoptotic effect [38, 39].

## Conclusion

Mixed neuroendocrine-non-neuroendocrine tumors localized in the biliary tract are extremely rare. This type of lesions can occur in any organ that has an embryonic origin from the primitive gut. The origin of these malignancies is still not fully understood. These tumors do not have specific symptoms, and this is the main reason they are often diagnosed in late stages of the disease. Spiral CT scanning is the method of choice for diagnosis and staging of the disease. The primary treatment modality should be radical surgery. Systemic chemotherapy, optimally combined with biological therapy with somatostatin analogues, has an indispensable role in the therapeutic algorithm.
